# Modeling Al-Qaraqoul canal before and after rehabilitation using HEC-RAS

**DOI:** 10.1038/s41598-024-63995-9

**Published:** 2024-06-26

**Authors:** Sara Zahran, Essam A. Gooda, Nesma AbdelMeged

**Affiliations:** Department of Irrigation and Hydraulics, Faculty of Engineering, Alexandria University, Alexandria, Egypt

**Keywords:** Irrigation canals, HEC-RAS, Qaraqoul canal, Water resources, Rehabilitation, Mesqa’s, Civil engineering, Hydrology, Engineering

## Abstract

The Egyptian Ministry of Water Resources and Irrigation launched in 2020 the national project to rehabilitate the canals network to rationalize the use of water resources to face the scarcity problems. The aim of study is to evaluate the impact of canal rehabilitation on the performance of irrigation water delivered laterally to Mesqa’s and longitudinally to the end of canal. Qaraqoul Canal et al.-Mallah Area, Alexandria, Egypt, was modeled using Hydrologic Engineering Center's-River Analysis System (HEC-RAS) to simulate water levels in the canal before and after rehabilitation using four discharge scenarios: 1.82, 3.7, 2.2, 7.87 m^3^/s. The calibration before rehabilitation shows that HEC-RAS simulated water levels corresponding to a discharge of 2.2 m^3^/s were in a good agreement with the actual field measured water levels. HEC-RAS results demonstrated that rehabilitation hydraulically improved the efficiency and performance of water conveyed by the canal. On the other hand, second scenario can be considered as suitable to keep water to reach the canal downstream with minimum suitable discharge, providing the need of two emergency pumps at last two branch canals called Mesqa’s. An ideal cross-section is also simulated using HEC-RAS which produced an efficient alternative with 40% less cost than the constructed alternative.

## Introduction

The existing irrigation canals in the Nile Delta were constructed nearly 100 years ago prior to the construction of Aswan High Dam^1^. As the Egyptian population increased and so the extension of irrigation areas, the water availability per feddan dropped significantly. The major water management policies are now largely involved with programs of rehabilitation rather than the construction of new irrigation infrastructures.

In the Nile Delta, irrigation water was usually pumped through a huge network of canals. The network consists of main, branch, tertiary canals called (Mesqa), and field ditches called Marwa^2^. Due to the weak annual maintenance^3^, clay deposits as well as weeds growth were widespread on both bed and side slopes of canals. Therefore, the rehabilitation of canals was urgent to reshape the existing cross and longitudinal sections of canals.

The main objectives of rehabilitation are summarized in removing all encroachments and irregularities on the berm level of canal to save sides slopes from collapsing due to lateral pressures, also distributing equal quantities of water to reach the ends of small canals without any sources of pollution resulting from irregularities and lifting machines. In addition to, rehabilitation aimed to save water losses and keep water to reach the end of the canals. Good quality of water is important to increase the agricultural production, efficiency of irrigation water to address climate change related water shortage, and reducing substantial losses of irrigation water caused by seepage from canals^4^.

The national program for the Rehabilitation of Irrigation Canals was launched by the President of Egypt in 2020^5^. Since then, the program has spread to all governorates. The works are implemented by fast-track procedures which mainly involve the following: Rehabilitation of canals by using pitching, as well as unreinforced and reinforced concrete, to improve the canal profile. It was also include the rehabilitation of bridges on the canal.

Mustafa^6^ studied water shortage at the ends of El-Bateekh canal and also studied the rehabilitation of the canal using the CCHE-GUI 3.29 program to improve performance and conveyance efficiency, reliability and durability of the system, which concluded the inefficiency of the canal to deliver the required discharges, however both of rehabilitation and remodeling the canal improved conveyance efficiency for the same discharge. Tawfiky^7^ studied Al-Rayah Al-Tawfiky which is a part of Damietta branch from Nile River. He applied different rehabilitation scenarios which were simulated using SOBEK1D model. It was concluded that implementing the rehabilitation works increase the capacity of Al-Rayah Al-Tawfiky by 25%, and 22.5% of actual inlet discharge for the fixed W.L case and the variable case respectively. This improving the capacity to be 97.01%, and 95.07%) respectively of the designed inlet discharge. Abd-Elziz^8^ evaluated the impact of Irrigation Canals Rehabilitation (ICR) using concrete on the land and its effect on crop yields for three canals in the Nile Delta, Egypt as a case study : Sero, Dafan, and New-Aslogy. The results demonstrate that ICR has decreased the losses from canals which resulted in lowering the groundwater. In addition, the water table underneath the embankment was lowered. Decreasing the groundwater table could help to protect the land from logging and increase crop yields. Talaat ElGamal^9^ studied the effect of irrigation network rehabilitation on water management-case study: Tanta Navigation canal—Egypt, he used the HEC-RAS simulation model to investigate the problem and propose feasible solutions, to convey the required water supply to the second reach of El-Kased canal. The results showed that the dredging of Dalel El-Kased canal improved the water convey efficiency and hydraulic performance of the canal enabling maintaining high water levels upstream Sorad regulator. On the other hand, constructing a new regulator at km 18.5 is another solution to solve the irrigation problem in El-Kased canal.

The present work is intended to study an existing canal which is called Qaraqoul canal. The existing cross and longitudinal sections before rehabilitation were suffering a shortage of water, especially at the end of canal. In the present study, a process of rehabilitation was suggested and performed. The new data of cross sections were modified and simulated by using HEC-RAS software to show how the canal performance was improved to allow water to distribute and reach the end of canal.

## Methodology

After deep search, it was found out that the use of HEC-RAS^10^ will be ideal software to do the simulation model in order to represent the existing situation as well as the suggested modifications after rehabilitation.

HEC-RAS is an integrated system of software, designed for interactive use in a multi-tasking environment. The software is used to study river analysis in case of different conditions such as steady flow water-surface profile computations, unsteady flow simulation, movable boundary sediment-transport computation, and water quality analysis. The current study uses the steady flow component. The input data included the canal geometric data (the actual surveyed cross-sections and the control structures of Mesqa’s in take at different locations in the canal), Chosen discharges based on different scenarios initial and boundary conditions.

The initial condition is the case of canal before rehabilitation where the canal was suffering from severe deterioration in the cross- section and fluctuation in bed levels of canal.

In addition to collapses in the side slopes and bed levels to affect the type of soil and consequently the Manning’s coefficient which its value varies before rehabilitation.

All collected data were used in HEC-RAS model as input data. As shown in flowchart for the methodology of case study.
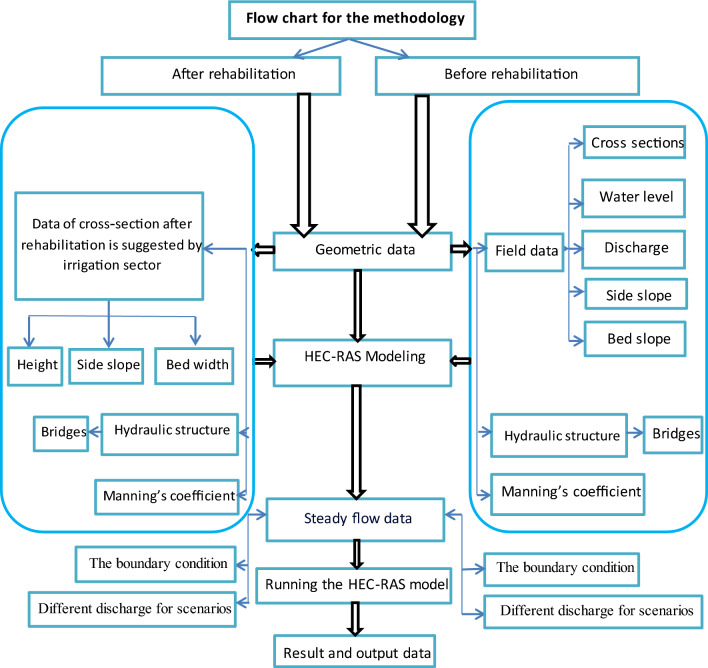


### Study area

**Figure 1:**
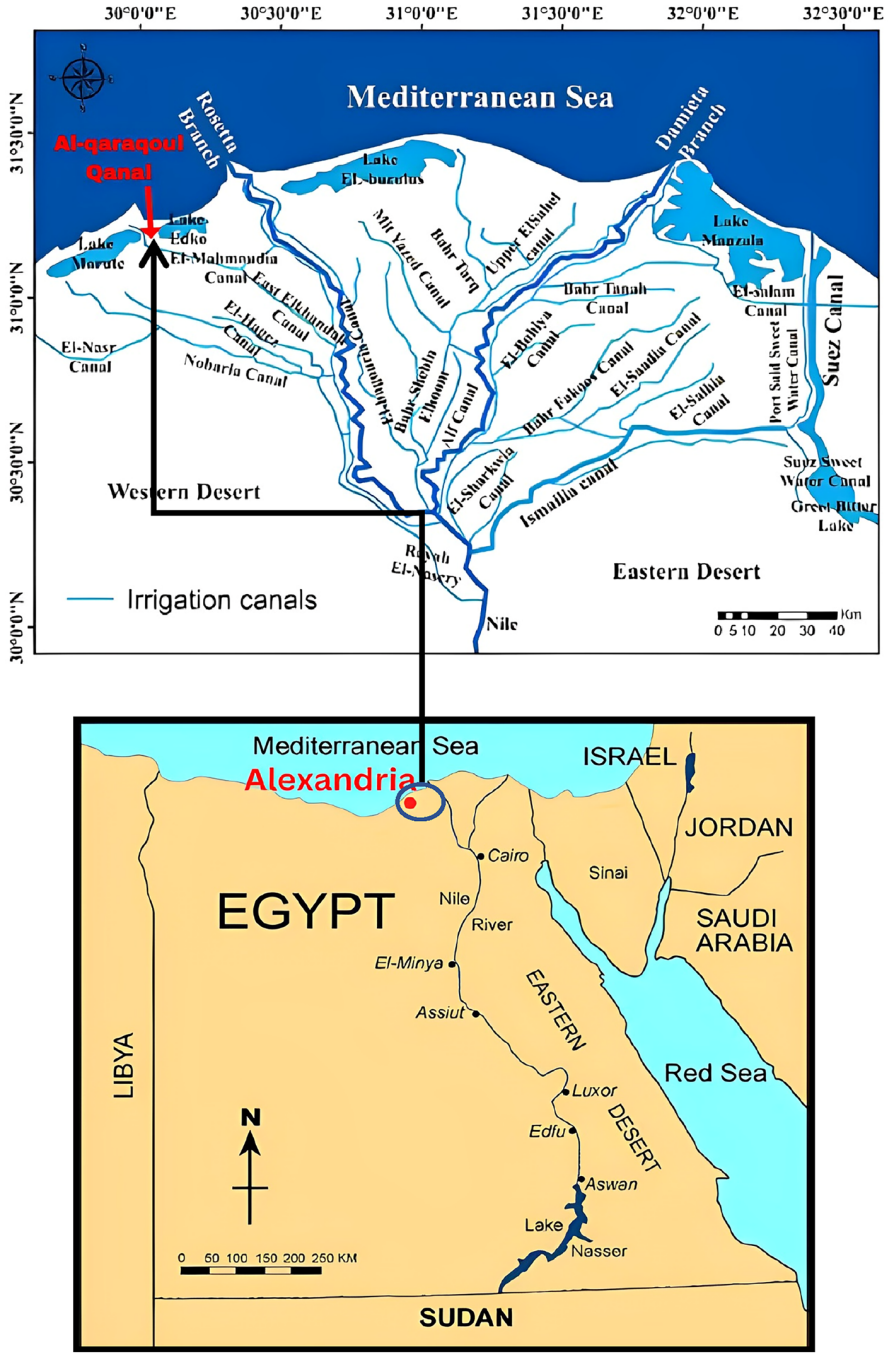
Qaraqoul canal at Al-Mallah area, Alexandria Governorate.

The ongoing research program of the Faculty of Engineering, Alexandria University aims to support and take part in national projects related to saving water and improving the irrigation system to maximize the role of Alexandria University in serving the society around, therefore a case study, Al Qaraqoul canal, as shown in Fig. 1, Alexandria Governorate was selected to be studied. Figure 1 which divided into two parts. Part 1 for the map is for the upper part of Egypt. It is common map^11^, it was used to show the location of canal for the governorates of Egypt. The second map is showing the location of the canal (case study) in the Egyptian canals Network and obtained from the General Authority for Survey of the Ministry of Water Resources and Irrigation of Egypt^12^, where I work as a civil engineer in the same ministry and the maps are drawn in the Ministry with a program (ARC MAP software).

Arc Map is the former main component of Esri's ArcGIS suite of geospatial processing programs. Used primarily to view, edit, create, and analyze geospatial data.

**Figure 2:**
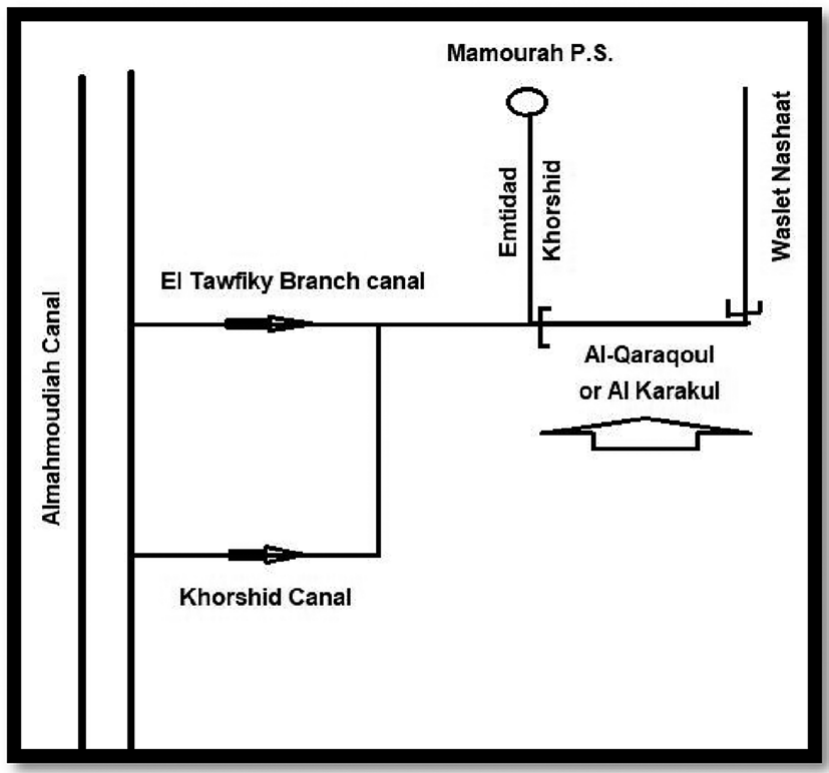
Schematic Plan showing main and secondary canals ending with Al-Qarqoul canal.

Al Qaraqoul Canal is located at Al-Mallah area, Alexandria Governorate that off-takes form Khorshid and El Tawfiky branch canals starting at Mahmoudia Main Canal, as shown in Fig. 2. This area extends approximately between 31°16′18.92ʺ N and 30°3′25.61ʺ E.

**Figure 3:**
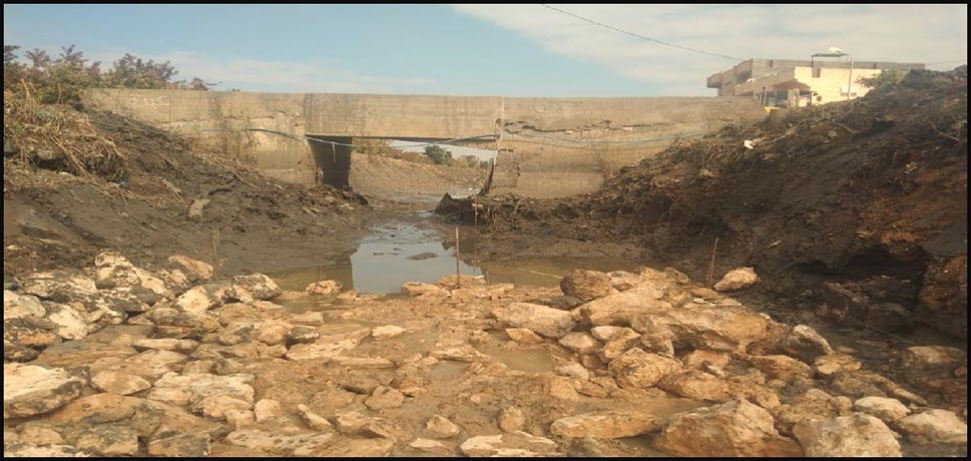
Illegal bridge at sec 2.0 km in Al-Qarqoul canal.

Al-Qaraqoul Canal serves about 4000 feddan^13^. There are two bridges located on the canal constructed by the Ministry of Irrigation. The canal is 6500 m long, part of the canal has been rehabilitated and the other part is covered, and bed width reduced from 4.0 to 2.0 m. The original side slopes were 1:1. The level of the bed of canal invert dropped from −2.75 to −3.05 m referred to The Egyptian Ministry of Water Resources and Irrigation (MWRI) datum. At the entrance, a head regulator exists to control the passing discharge of water entering the canal according to the agriculture requirement. The canal has several unsafe illegal buildings and small wooden and concrete bridges obstructing the flow of water as shown in Fig. 3**,** which may cause side slope collapses.

The violating bridges that were built on the canal, which prevented the passage of water and caused sedimentation of the soil, were removed. Only one bridge was constructed, with a design that would allow the passage of water to the ends of canals, and hence the water level would be sufficient for all Mesqa’s.

**Figure 4:**
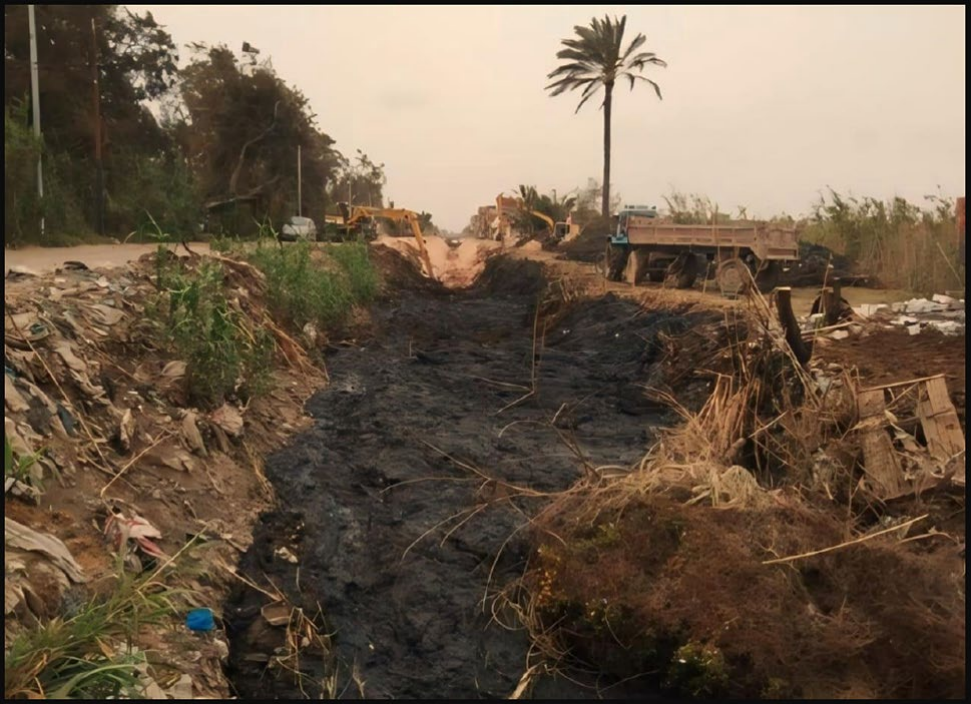
Sedimentation in canal at sec (1000 m).

**Figure 5:**
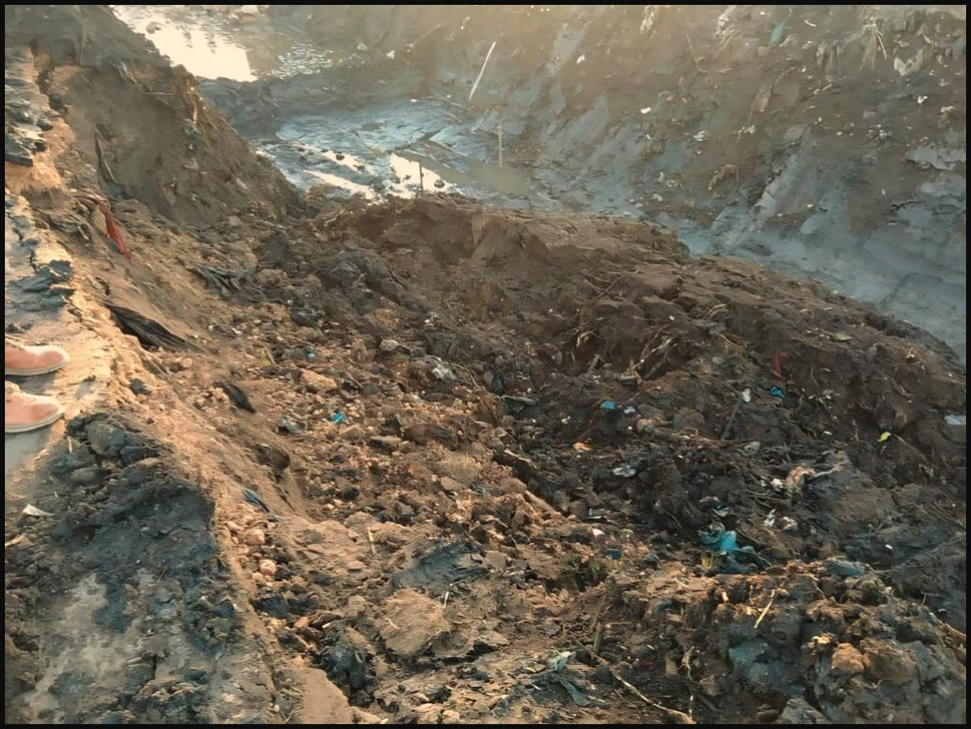
Collapses sides slopes at sec (1900 m).

There are also many problems in the canal responsible for the lack of water reaching the end and the quality of the water such as sedimentation and side slope collapses, as shown in Figs. 4 and 5. The figures also show that the canal reached a high level of deterioration and there is an urgent need for rehabilitation to provide safe and stable water supply and prevent flooding of lands.

### Modeling of Al-Qaraqoul canal before rehabilitation

Data was collected for Al-Qaraqoul canal based on Ministry of agriculture and the Ministry of water resources and irrigation (MWRI). This data include cultivated crops throughout the year as well as water need, actual area of agricultural lands and the corresponding discharge delivered by the canal.

At the site, locations and levels of illegal bridges on the canal as well as the cross and longitudinal sections were surveyed showing levels of bed, berm and water. Also, data includes, side slope and type of soil to determine the Manning’s coefficient value representing the canal. In addition, the levels of Mesqa’s inlet pipes were surveyed. HEC-RAS model is dependent on a set of data that include geometry of canal, discharge and conditions for the flow boundaries of the canal, etc. All collected data were used in HEC-RAS as input data. Several discharge scenarios were applied under certain assumptions (Q = 1.82, 3.7, 2.2, 7.87 m^3^/s). Al-Qaraqoul canal is illustrated in 15 cross-sections; Fig. 7 shows illustration section at 1500 m.

**Figure 6:**
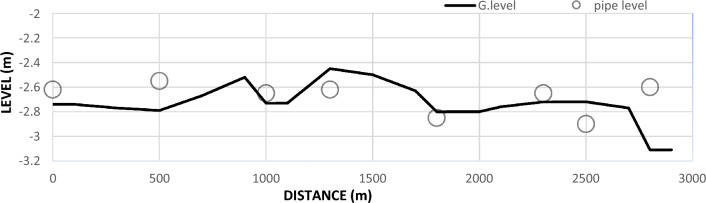
Mesqa’s pipe level with relative to canal bed level.

**Figure 7:**
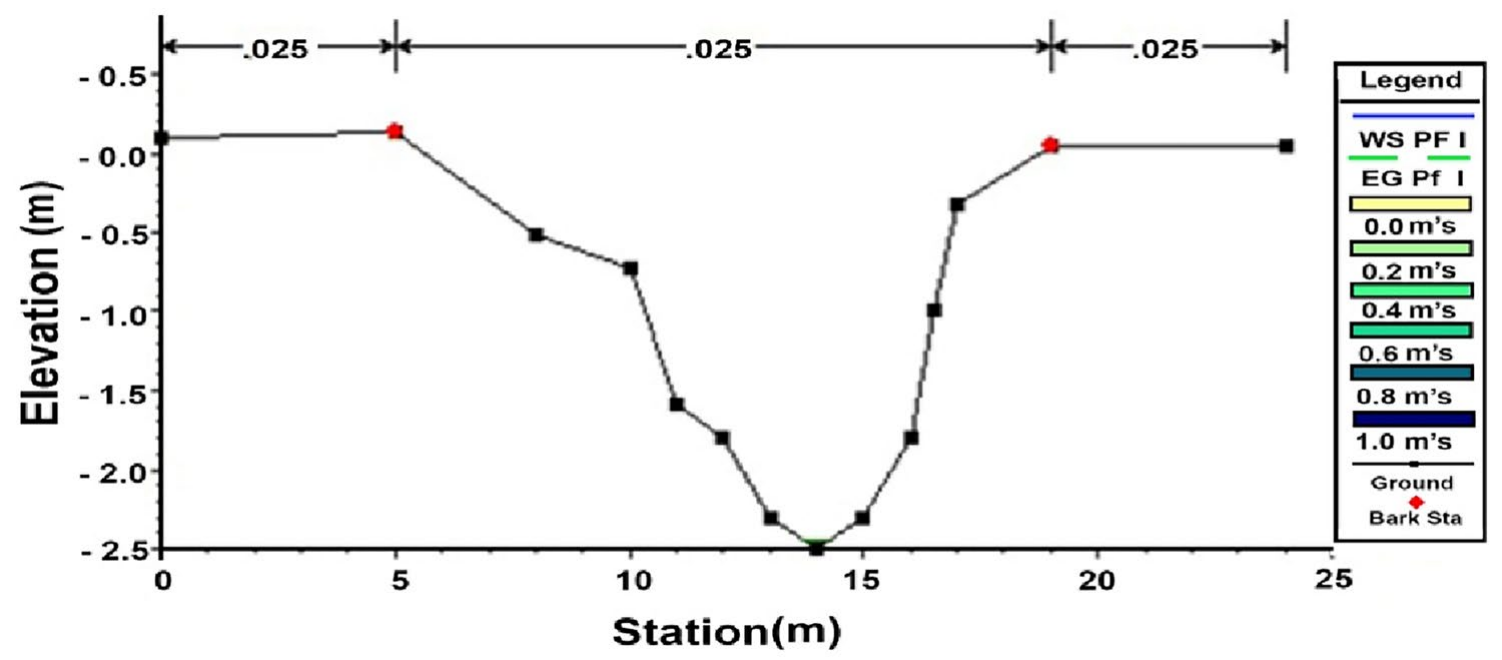
Cross section plotted in HEC-RAS before rehabilitation at sec 1500 m.

Due to bad maintenance, the accumulated sedimentation since the construction of canal, the bed levels were observed to be higher than the levels of some inlets of Mesqa’s pipes, as shown in Fig. 6. This was responsible for the shortage of irrigation water to feed Mesqa’s pipe (Fig. 7).

The first and second discharges, respectively, represent the past and current situation of agricultural lands at both sides of the canal. The third discharge is based on the actual amount of water related to the actual gate opening in nature, while the fourth is based on the calculated discharge after canal rehabilitation using Manning’s equations given in Table 1.

**Table 1:** Discharges calculated in each scenario.

Scenario	Discharge m^3^/s	Chosen discharges based on different scenarios
First	1.82	Based on served area of 1965 feddans and the time of irrigation is assumed 12 h/day
Second	3.7	Based on served area of 4000 feddans and the time of irrigation is assumed 12 h/day
Third	2.2	The proposed discharge is based on the actual amount of water in the nature .Discharge of 2.2 is considered as the method of calibration process. As shown in Fig. 11
Fourth	7.87	Applying Manning equation to give the discharge of canal cross-section. (b = 4 m, y = 1.94 m, z = 1, S = 10 cm/km, n = 0.0167)

### Al-Qaraqoul canal data after rehabilitation

**Figure 8:**
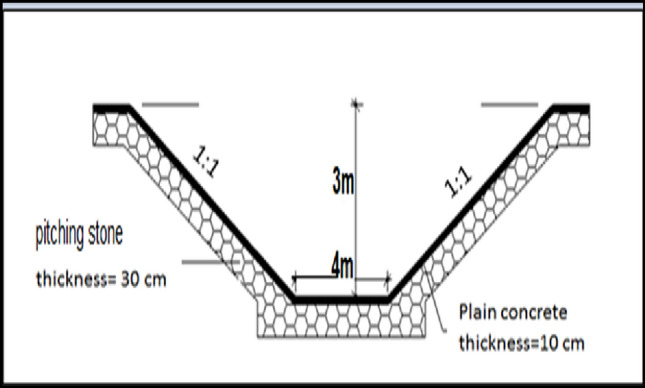
The design cross-section of the Qarqoul canal after rehabilitation.

**Figure 9:**
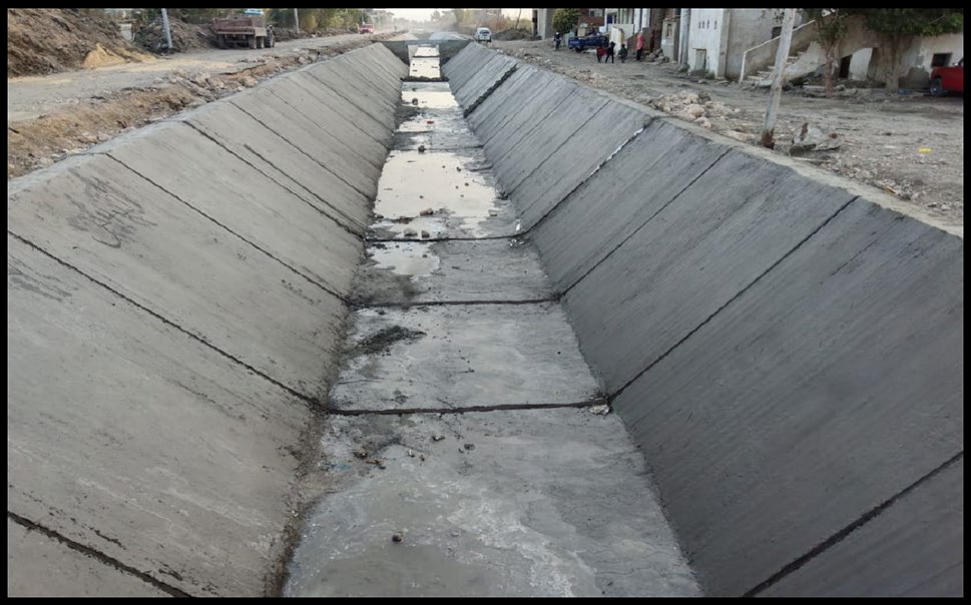
Lining works in the canal after rehabilitation.

The rehabilitation works were carried out for a length of 3000 km, using specifications done by the MWRI in the implementation of the rehabilitation of canals depending on type of soil. The procedures include:

- Excavation works to reach the design levels of bed and berm of canal.
- Well compaction to soil surface along the side slopes of 1:1 according to soil type.
- After rehabilitation, the bed width of canal changed at different sections along the canal at sec (3000, 1500 and 500 m) from 4 to 2 m depending on design data from MWRI^14^.
- Determining the locations of legal bridges on the canal and determining the levels for each bridge.
- Rehabilitation works start by using stone pitching of thickness 30 cm.
- A layer of plain concrete with a thickness of 10 cm is maintained on side slopes of 1:1 as shown Fig. 8.
- In addition, expansion joints at a distance of 16 m and contraction joints at a distance of 4 m are performed as shown in Fig. 9. Joints are filled and treated with bitumen.

**Figure 10:**
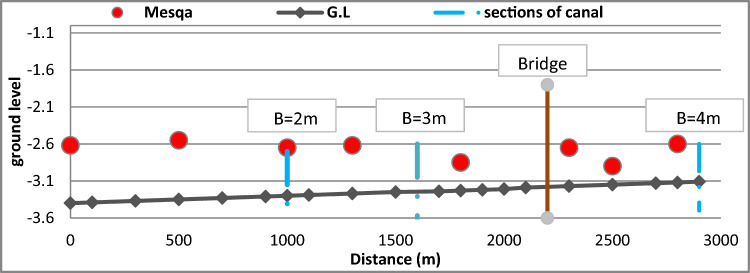
Longitudinal section for Qarqoul canal after rehabilitation.

Figure 10 shows the longitudinal section for Qarqoul canal depending on design data from MWRI after rehabilitation, Where the implementation of the cross-section of canal began with a bed width 4 m and side slopes 1:1 for a distance of 1500 m, then the bed width of the canal was reduced from 4 to 3 m at a distance of 500 m to reach the 1000 m along the canal and then bed width was completed by 1 m for the end of the part to be rehabilitated from the canal for a distance of 1000 m.

## Results and discussions

### Calibration of water levels measured before rehabilitation

**Figure 11:**
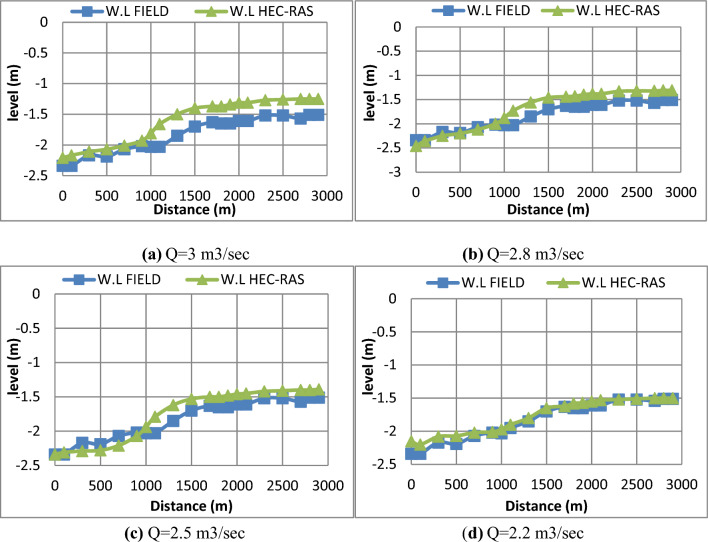
Comparison of water levels measured at the field with HEC-RAS output results. (**a**) Q = 3 m^3^/s, (**b**) Q = 2.8 m^3^/s, (**c**) Q = 2.5 m^3^/s, (**d**) Q = 2.2 m^3^/s. (**d**) Complete matching between measured and simulated water levels.

It is common to control the passing discharge in the canal through an intake structure such as a head regulator. In the field, there are no means of measuring the passing discharge, however, in the current study, the passing discharge was calibrated through measuring the water levels. For this purpose, water levels were measured in the canal before rehabilitation and several discharge values were assumed. For each discharge value, a simulation model was used to check each scenario. HEC-RAS output results of water levels were compared with actual measured values. At a complete matching between simulated and measured values of water levels, the assumed discharge was confirmed as a measured value. The selected discharges are shown in Fig. 11**.** As shown in Fig. 11d, the discharge value of 2.2 m^3^/s confirmed the required complete matching.

### Results of modeling scenarios before and after canal rehabilitation

Several discharge values based on the selected scenarios as referred earlier in Table 1, were modeled using HEC-RAS software to model flow and check the rehabilitation scenarios.

#### (a) The First scenario

**Figure 12:**
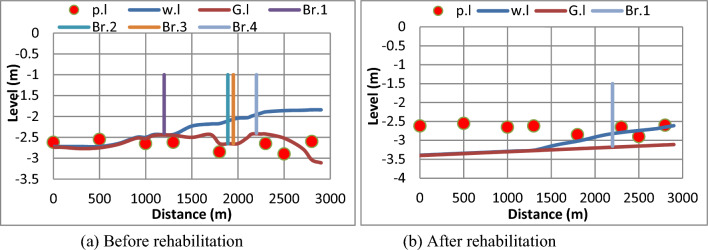
Water levels along the canal and at Mesqa’s pipe inlets before and after canal rehabilitation for first scenario. (**a**) Before rehabilitation, (**b**) after rehabilitation.

The First scenario is based on served area of 1965 feddans and the time of irrigation is assumed 12 h/day. The output is shown in Fig. 12, confirmed that before and after rehabilitation, the discharge of 1.82 m^3^/s was not enough to reach the end of the canal.

Initially, before the rehabilitation, the Mesqa’s pipe inlets at stations; 2500, 2300, 1800, 1300 and 1000 m, were placed under the ground levels due to the sedimentation, as shown in Fig. 12a. Therefore, water did not reach the end of canal, and so there was no water delivered to the Mesqa’s inlet at stations; 0 and 500 m. Furthermore, illegal bridges obstructed the water flowing in the canal since part of the bridge was constructed in the middle of water section as referred earlier in Fig. 3**.** On the other hand, after rehabilitation, the presence of the bridge did not affect the movement of water through the canal. Finally, after rehabilitation, the canal gained hydraulic characteristics, longitudinal slope, bed width, side slopes and bed levels as shown in Fig. 12b.

#### (b) The second scenario

**Figure 13:**
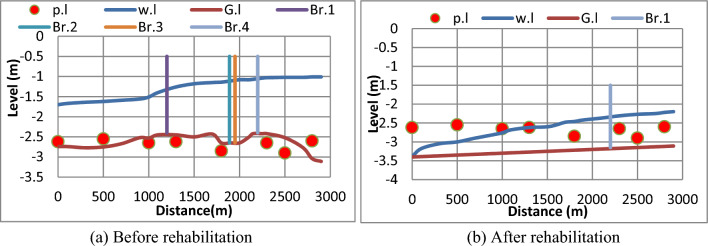
Water levels along the canal and at Mesqa’s pipe inlets before and after canal rehabilitation for the second scenario. (**a**) Before rehabilitation, (**b**) after rehabilitation.

The second scenario is based on served area of 4000 feddans and the time of irrigation is assumed 12 h/day. The output is shown in Fig. 13, It has been confirmed for the second scenario that before rehabilitation, the discharge of 3.7 m^3^/s reached the end of canal as shown in Fig. 13a. The flow did not reach all Mesqa’s, because the Mesqa’s pipe inlet was placed under the ground level due to sedimentation.

On the other hand, the water depths were improved after rehabilitation to cover the entrances of Mesqa’s pipe inlet except the last three Mesqa’s which were in need of small pump units to raise the water levels, as shown in Fig. 13b.

#### (c) The third scenario

**Figure 14:**
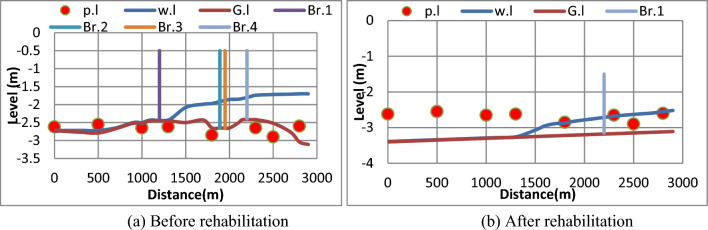
Water levels along the canal and at Mesqa’s pipe inlets before and after canal rehabilitation for the third scenario. (**a**) Before rehabilitation, (**b**) after rehabilitation.

The third scenario is based on the actual amount of water discharge related to the actual gate opening in nature. It has been noticed that before rehabilitation the discharge 2.2 m^3^/s was not enough to reach some Mesqa’s pipe inlet which led to a lack of water to irrigate agricultural lands as shown in Fig. 14a. After rehabilitation, it has been confirmed that the discharge was not enough to reach the end of the canal, as shown in Fig. 14b.

#### (d) The fourth scenario

**Figure 15:**
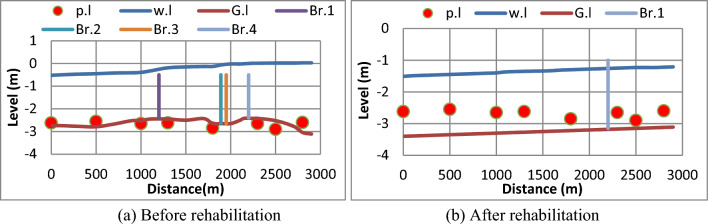
Water levels along the canal and at Mesqa’s pipe inlets before and after canal rehabilitation for the fourth scenario. (**a**) Before rehabilitation, (**b**) after rehabilitation.

The fourth scenario is based on applying Manning equation to give the discharge of canal cross-section. (b = 4 m, y = 1.94 m, z = 1, S = 10 cm/km, n = 0.0167). It has been shown that before rehabilitation, when applying the mentioned large discharge 7.87 m^3^/s, a flood occurred in the canal as shown in Fig. 15a. Thus, overtopping has occurred in the canal sections adjacent to the lands served by the canal. After rehabilitation, the water depth was improved to cover all Mesqa’s pipe inlet to meet the required water levels in order to enter the Mesqa’s pipe by gravity without the need of pump as shown in Fig. 15b.

After applying the different scenarios, it has been observed from the previous graphs that the water surface before rehabilitation fluctuated in all scenarios due to the illegal bridges, the instability of bed levels with varying sedimentation at the bed of canal. This has led to an increase in the water levels.

After rehabilitation, it was observed that the water levels were stable and affected by the passing discharge and the canal bed slope. Therefore, the water levels decreased compared to that before rehabilitation. In addition to increase in the water velocity in the canal, which led to a reduction in the period of irrigation of the reins located on the canal and the time of operation of the irrigation pumps, and a reduction in the annual expenditure on maintenance and purification of the canals.

For large discharge based on the canal design section at the present time, the ideal cross-section should be implemented.

## Ideal section of Al-Qarqoul canal after rehabilitation

After rehabilitation, it was found that the discharge passing through the canal gate was large and was considered as wasted water since it was more than double the discharge required by the surrounding agricultural lands. Hence, reducing the dimensions of canal led to saving large amounts of water as well as large amounts of concrete used in the rehabilitation process. Thus, the ideal section must be implemented.

**Figure 16:**
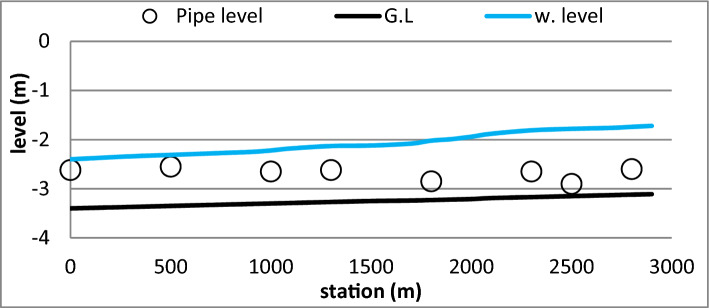
Water surface profile for the ideal section.

The ideal section of canal is considered more economical and efficient in delivering water. Therefore, attempts were made to have an ideal and suitable cross-section. bed width 2.5 m, side slope 1:1, and water level 1.8 m. Keeping the side slopes and the roughness coefficient, it was concluded that the discharge 4.7 m^3^/s was the optimal discharge, and the water level was also sufficient for all Mesqa’s, as shown in Fig. 16.

## Conclusions

Since the national program for Rehabilitation of Irrigation Canals was launched by the President of Egypt in 2020 and implemented by fast-track procedures, several studies were done to evaluate the effect of rehabilitation on the efficiency of water movement. The current study discusses the effect of rehabilitation on the canal before and after. Initially problems facing the canal before rehabilitation such as seepage, sedimentation, and side slope collapses, were eliminated by lining the canal with concrete.

Therefore, after rehabilitation, there was no seepage as well as no soil sedimentation. Moreover, Mesqa’s pipes became free of sedimentation which was responsible for blockage and preventing water to reach so many agricultures area. Rehabilitation had a good effect on the efficiency of water movement laterally and longitudinal.

In the current methodology, HEC-RAS was found to be the best software to model and simulate the case study of Al-Qaraqoul canal, Al-Mallah area, Alexandria, Egypt, before and after rehabilitation.

HEC-RAS model was selected and calibrated to simulate water levels in Qaraqoul canal using four discharge scenarios: 1.82, 3.7, 2.2, and 7.87 m^3^/s running before and after rehabilitation.

Calibration performed before rehabilitation showed that HEC-RAS output simulated water levels corresponding to a discharge of 2.2 m^3^/s was in good agreement with actual water levels measured in the field. Also, HEC-RAS output results obtained in the current study demonstrated that after rehabilitation, the discharge of 7.87 m^3^/s was considered an efficient discharge to deliver water laterally to all Mesqa’s as well as longitudinally to the end. However, it was considered as wasted water, since it represents more than double the discharge required by the surrounding agricultural lands.

Therefore, the current study suggested having an ideal canal cross-section to reduce the dimensions of canal to save a large amount of both water and concrete lining. The design of an ideal cross-section, b = 2.5m, y = 1.8 m, z = 1, S = 10 cm/km, n = 0.0167, was responsible for 40% reduction in the cost of construction. Additionally, an ideal cross-section of canal was also simulated to be considered as economical and efficient in delivering water.

Finally, it has been concluded that the second scenario of 3.7 m^3^/s can be considered as suitable to keep the water reach downstream of Qaraqoul Canal under good condition except the need of small emergency pumps at the last two Mesqa’s pipes.

## Data Availability

The on-site data was collected in compliance with the Ministry of Water Resource and Irrigation project specification, in addition to ministry of agriculture, Egypt. Further, any additional data can be obtained upon request by sending an email to the Correspondence author: sara.zahran19@alexu.edu.eg. The collected data are available at the following links: https://www.mwri.gov.eg/ and https://moa.gov.eg/en/.
