# Author Correction: Modeling Al-Qaraqoul canal before and after rehabilitation using HEC-RAS

**DOI:** 10.1038/s41598-024-74721-w

**Published:** 2024-10-04

**Authors:** Sara Zahran, Essam A. Gooda, Nesma AbdelMeged

**Affiliations:** Department of Irrigation and Hydraulics, Faculty of Engineering, Alexandria University, Alexandria, Egypt

Correction to: *Scientific Reports*10.1038/s41598-024-63995-9, published online 26 June 2024.

In the original version of this Article Sara Zahran, Essam A. Gooda and Nesma AbdelMeged were omitted as equally contributing authors. The statement now reads:

“These authors contributed equally: Sara Zahran, Essam A. Gooda and Nesma AbdelMeged.”

The original Article has been corrected.

